# Predictors of acute kidney injury after paraquat intoxication

**DOI:** 10.18632/oncotarget.17975

**Published:** 2017-05-18

**Authors:** Cheng-Hao Weng, Hui-Hsiang Chen, Ching-Chih Hu, Wen-Hung Huang, Ching-Wei Hsu, Jen-Fen Fu, Wey-Ran Lin, I-Kwan Wang, Tzung-Hai Yen

**Affiliations:** ^1^ Department of Nephrology and Poison Center, Chang Gung Memorial Hospital and College of Medicine, Chang Gung University, Linkou, Taiwan; ^2^ Department of Hepatogastroenterology and Liver Research Unit, Chang Gung Memorial Hospital, Keelung, Taiwan; ^3^ Department of Medical Research, Chang Gung Memorial Hospital and College of Medicine, Chang Gung University, Linkou, Taiwan; ^4^ Department of Gastroenterology and Hepatology, Chang Gung Memorial Hospital and College of Medicine, Chang Gung University, Linkou, Taiwan; ^5^ Department of Nephrology, Chang Medical University Hospital and College of Medicine, China Medical University, Taichung, Taiwan; ^6^ Kidney Research Center, Chang Gung Memorial Hospital, Linkou, Taiwan; ^7^ Center for Tissue Engineering, Chang Gung Memorial Hospital, Linkou, Taiwan

**Keywords:** paraquat, suicide, acute kidney injury, SOFA, AKIN

## Abstract

Paraquat intoxication is characterized by multi-organ failure, causing substantial mortality and morbidity. Many paraquat patients experience acute kidney injury (AKI), sometimes requiring hemodialysis. We observed 222 paraquat-intoxicated patients between 2000 and 2012, and divided them into AKI (*n* = 103) and non-AKI (*n* = 119) groups. The mortality rate was higher for AKI than non-AKI patients (70.1% vs. 40.0%, *P <* 0.001). Patients with AKI had a longer time to hospital arrival (*P* = 0.003), lower PaO_2_ (*P* = 0.006) and higher alveolar-arterial O_2_ difference (*P <* 0.001) 48 h after admission, higher sequential organ failure assessment 48-h score (*P <* 0.001), higher severity index of paraquat poisoning (SIPP) score (*P* = 0.016), lower PaCO_2_ at admission (*P* = 0.031), higher PaO_2_ at admission (*P* = 0.015), lower nadir PaCO_2_ (*P* = 0.001) and lower nadir HCO_3_ (*P* = 0.004) than non-AKI patients. Multivariate logistic regression indicated that acute hepatitis (*P <* 0.001), a longer time to hospital arrival (*P <* 0.001), higher SIPP score (*P* = 0.026) and higher PaO_2_ at admission (*P* = 0.014) were predictors of AKI. The area under the receiver operating characteristic curve confirmed that an Acute Kidney Injury Network 48-hour score ≥ 2 predicted AKI necessitating hemodialysis with a sensitivity of 0.6 and specificity of 0.832. AKI is common (46.4%) following paraquat ingestion, and acute hepatitis, the time to hospital arrival, SIPP score and PaO_2_ at admission were powerful predictors of AKI. Larger studies with longer follow-up durations are warranted.

## INTRODUCTION

Paraquat is a contact herbicide with extremely high toxicity. Paraquat is easily accessible, and in Taiwan, it is commonly ingested, whether deliberately or unintentionally [1]. Due to its severe toxicity, paraquat consumption is fatal in 60–80% of cases. Patients who have consumed about 40 mL of a 24% paraquat solution typically die of multiple organ failure in the next few hours or days [2]. Upon ingestion, paraquat is absorbed quickly, although not completely, and is mostly excreted in the urine without further metabolism within 12–24 hours. Although the characteristic clinical features of paraquat intoxication are acute lung injury and multiple organ failure, acute kidney injury (AKI) also frequently occurs after acute large-dose exposure to this toxic herbicide [3].

Any patient who comes to our hospital with paraquat intoxication is treated with a standard detoxification protocol including charcoal hemoperfusion, pulse therapies with methylprednisolone and cyclophosphamide, and extended treatment with dexamethasone [4, 5]. For most patients, this practice nearly normalizes respiratory function and blood oxygen concentrations within three to six months [6]. This protocol has been reviewed and recommended by the Cochrane Injuries Group as beneficial in cases of lung fibrosis caused by paraquat [7]. Many paraquat patients experience AKI, and some may still progress to AKI necessitating hemodialysis (HD) despite intensive treatment.

The mechanism whereby paraquat causes AKI is not fully understood; however, it is known that this compound can accrue within renal tubular cells, leading to cycles of reduction and oxidation, generating reactive oxygen species, and ultimately damaging the proximal tubules [8]. As the kidney is the main organ responsible for paraquat excretion, the resultant kidney injury may reduce the elimination of paraquat and increase its toxicity in other organs [9]. Mohamed et al. [10] demonstrated that kidney damage biomarkers such as neutrophil gelatinase-associated lipocalin were upregulated in AKI, but did not independently predict death. Only functional markers such as serum creatinine predicted mortality after paraquat intoxication. Reduced kidney function is only one factor causing the rapid rise in creatinine after paraquat poisoning [11]. During severe oxidative stress, it is likely that higher levels of creatine and creatinine are generated to meet the increased need for energy. To a lesser degree, acidosis may increase creatine cyclisation to creatinine, or creatinine secretion may be competitively or non-competitively inhibited.

Kim et al. [12] reported that hyperuricemia increased the risk of mortality 3.7-fold and increased the risk of kidney failure 3.3-fold (adjusting for age, sex and estimated paraquat intake), and proposed that the serum level of uric acid was a reliable clinical predictor of death or AKI in patients with acute paraquat poisoning. In our previous studies [13, 14], we did not identify a strong correlation between the serum uric acid level and mortality. Theoretically, kidney failure reduces the elimination of uric acid and might increase serum uric acid levels.

In this study, we investigated the clinical features, AKI spectrum and outcomes in patients with deliberate paraquat intoxication, and most importantly, we evaluated the predictors of AKI after paraquat intoxication.

## RESULTS

### Subject characteristics

As shown in Table 1, the mortality rate was higher for AKI patients than for non-AKI patients (70.1% vs. 40.0%, *P <* 0.001). Furthermore, AKI patients exhibited a longer time to hospital arrival (17.7 ± 24.5 vs. 9.9 ± 17.1 hours, *P* = 0.003), lower PaO_2_ 48 hours after admission (56.0 ± 26.0 vs. 65.9 ± 23.0 mmHg, *P* = 0.006), higher alveolar-arterial oxygen gradient (AaDO_2_) 48 hours after admission (56.1 ± 27.9 vs. 38.4 ± 26.2 mmHg, *P <* 0.001), higher SOFA_48h_ score (4.7 ± 2.1 vs. 1.8 ± 1.5, *P <* 0.001), higher SIPP score (0.7 ± 0.5 vs. 0.5 ± 0.5, *P* = 0.016), lower partial pressure of arterial carbon dioxide (PaCO_2_) at admission (31.0 ± 10.5 vs. 34.2 ± 8.1 mmHg, *P* = 0.031), higher PaO_2_ at admission (88.5 ± 20.4 vs. 81.8 ± 17.1 mmHg, *P* = 0.015), lower nadir PaCO_2_ (30.1 ± 9.0 vs. 36.8 ± 14.0 mmHg, *P* = 0.001) and lower nadir bicarbonate (HCO_3_) (18.3 ± 7.3 vs. 21.3 ± 6.0 mmHg, *P* = 0.004) than non-AKI patients.

**Table 1:** Comparisons between paraquat patients with and without AKI (*n* = 222)

	AKI (*n* = 103)	Non-AKI (*n* = 119)	*P*
Age	41.8 ± 15.9	42.3 ± 15.1	0.825
Gender (male), *n* (%)	86 (83.9%)	86 (72.0%)	0.056
Mortality, *n* (%)	72 (70.1%)	48 (40.0%)	< 0.001***
ARDS, *n* (%)	33 (32.2%)	29 (24.0%)	0.253
Acute lung injury, *n* (%)	64 (62.1%)	48 (40.0%)	0.479
Pneumomediastinum, *n* (%)	8 (7.8%)	0	0.002**
Time to hospital arrival (hours)	17.7 ± 24.5	9.9 ± 17.1	0.003**
Estimated ingestion amount (mL)	80.8 ± 77.3	81.0 ± 124.1	0.987
Blood paraquat concentration (ppm)	4.6 ± 4.6	4.9 ± 6.4	0.731
Peak ALT concentration (U/L)	190.9 ± 161.3	176.5 ± 69.5	0.442
Peak AST concentration (U/L)	122.4 ± 82.8	132.9 ± 247.8	0.909
Peak bilirubin concentration (mg/dL)	3.31 ± 2.1	3.0 ± 1.2	0.279
Nadir PaO_2_ (mmHg)	56.0 ± 26.0	65.9 ± 23.0	0.006**
Nadir AaDO_2_(mmHg)	56.1 ± 27.9	38.4 ± 26.2	< 0.001***
SOFA_48h_	4.7 ± 2.1	1.8 ± 1.5	< 0.001***
SIPP score (hours/mg/L)	0.7 ± 0.5	0.5 ± 0.5	0.016*
PaCO_2_ at admission (mmHg)	31.0 ± 10.5	34.2 ± 8.1	0.031*
PaO_2_ at admission (mmHg)	88.5 ± 20.4	81.8 ± 17.1	0.015*
Nadir PaCO_2_(mmHg)	30.1 ± 9.0	36.8 ± 14.0	< 0.001***
Nadir HCO_3_ (mmHg)	18.3 ± 7.3	21.3 ± 6.0	0.004**

Note: *P < 0.05, **P < 0.01, ***P < 0.001, AKI acute kidney injury, ARDS acute respiratory distress syndrome, AaDO_2_ alveolar-arterial oxygen gradient, ALT alanine aminotransferase, AST aspartate aminotransferase, HCO_3_ bicarbonate, PaO_2_ partial pressure of arterial oxygen, PaCO_2_partial pressure of arterial carbon dioxide, SOFA sequential organ failure assessment, SIPP severity index of paraquat poisoning

In addition, the mortality rate tended to be higher for AKI HD patients than for non-AKI HD patients, although this trend was not statistically significant (80.0% vs. 50.9%, *P* = 0.107, Table 2). Nevertheless, the AKI HD group demonstrated a higher risk of ARDS (50.0% vs. 25.1%, *P* = 0.032), higher AaDO_2_ 48 hours after admission (58.5 ± 31.9 vs. 45.2 ± 27.7 mmHg, *P* = 0.047), higher SOFA_48h_ score (4.5 ± 2.5 vs. 3.0 ± 2.2, *P* = 0.007), higher SIPP score (0.9 ± 0.4 vs. 0.5 ± 0.5, *P* = 0.001), and lower PaCO_2_ at admission (28.2 ± 8.1 vs. 34.3 ± 12.6 mmHg, *P* = 0.034) than the non-AKI HD group.

**Table 2:** Comparisons between paraquat patients with and without AKI HD (*n* = 222)

	AKI HD (*n* = 24)	Non-AKI HD (*n* = 198)	*P*
Age	38.0 ± 10.9	42.6 ± 15.8	0.204
Gender (male), *n* (%)	17 (70%)	155 (78.4%)	0.401
Mortality, *n* (%)	19 (80%)	101 (50.9%)	0.107
ARDS, *n* (%)	12 (50.0%)	50 (25.1%)	0.032*
Acute lung injury, *n* (%)	14 (60.0%)	98 (49.4%)	0.479
Pneumomediastinum, *n* (%)	3 (12.5%)	5 (2.5%)	0.047*
Time to hospital arrival (hours)	15.0 ± 19.7	13.3 ± 21.3	0.744
Estimated ingestion amount (mL)	77.1 ± 82.8	81.4 ± 107.2	0.862
Blood paraquat concentration (ppm)	4.8 ± 3.5	4.8 ± 5.8	0.992
Peak ALT concentration (U/L)	239.5 ± 220.5	176.5 ± 102.1	0.221
Peak AST concentration (U/L)	189.5 ± 174.7	120.6 ± 139.1	0.508
Peak bilirubin concentration (mg/dL)	3.1 ± 0.7	3.2 ± 1.8	0.868
Nadir PaO_2_ (mmHg)	56.2 ± 34.1	61.9 ± 23.6	0.477
Nadir AaDO_2_(mmHg)	58.5 ± 31.9	45.2 ± 27.7	0.047*
SOFA_48h_	4.5 ± 2.5	3.0 ± 2.2	0.007**
SIPP score (hours/mg/L)	0.9 ± 0.4	0.5 ± 0.5	0.001**
PaCO_2_ at admission (mmHg)	29.3 ± 8.5	33.0 ± 9.5	0.169
PaO_2_ at admission (mmHg)	91.1 ± 20.5	84.2 ± 18.7	0.123
Nadir PaCO_2_ (mmHg)	28.2 ± 8.1	34.3 ± 12.6	0.034*
Nadir HCO_3_ (mmHg)	18.0 ± 6.6	20.1 ± 6.8	0.180

Note: *P < 0.05, **P < 0.01, AKI HD acute kidney injury necessitating hemodialysis, ARDS acute respiratory distress syndrome, AaDO_2_ alveolar-arterial oxygen gradient, ALT alanine aminotransferase, AST aspartate aminotransferase, HCO_3_ bicarbonate, PaO_2_ partial pressure of arterial oxygen, PaCO_2_ partial pressure of arterial carbon dioxide, SOFA sequential organ failure assessment, SIPP severity index of paraquat poisoning.

### Predictors of AKI

Multivariate logistic regression analysis demonstrated that acute hepatitis (*P <* 0.001), a longer time to hospital arrival (*P <* 0.001), higher SIPP score (*P* = 0.026) and higher PaO_2_ at admission (*P* = 0.014) were significant predictors of AKI (Table 3). The sensitivity, specificity and cutoff values of these predictors of AKI are summarized in Table 5.

**Table 3:** Analysis of AKI with univariate and multivariate logistic regression models (*n* = 222)

	B	SE	Exp (B)	*P*
Univariate				
Time to hospital arrival (hours)	0.019	0.008	1.019 (1.004–1.035)	0.015*
SIPP score (hours/mg/L)	0.722	0.302	2.058 (1.140–3.717)	0.017*
Acute hepatitis	1.740	0.322	5.697 (3.033–10.699)	< 0.001***
PaO_2_ at admission (mmHg)	0.020	0.008	1.020 (1.003–1.037)	0.018*
Nadir PaO_2_(mmHg)	-0.017	0.006	0.983 (0.971–0.996)	0.008**
Nadir PaCO_2_(mmHg)	-0.066	0.018	0.936 (0.904–0.969)	< 0.001***
Nadir HCO_3_(mmHg)	-0.065	0.023	0.937 (0.896–0.979)	0.004**
Nadir AaDO_2_(mmHg)	0.024	0.006	1.025 (1.013–1.037)	< 0.001***
Acute lung injury	0.881	0.301	2.414 (1.337–4.356)	0.003**
Multivariate				
Acute hepatitis	2.230	0.414	9.301 (4.835–25.462)	< 0.001***
Time to hospital arrival (hours)	0.045	0.010	1.046 (1.030–1.074)	<0.001***
SIPP score (hours/mg/L)	0.958	0.429	2.606 (1.066–6.010)	0.026*
PaO_2_ at admission (mmHg)	0.027	0.011	1.027 (1.008–1.053)	0.014*

Note: *P < 0.05, **P < 0.01, B beta coefficient or regression coefficient, Exp (B) exponential B or odds ratio, AKI acute kidney injury, ARDS acute respiratory distress syndrome, AaDO_2_ alveolar-arterial oxygen gradient, ALT alanine aminotransferase, AST aspartate aminotransferase, HCO_3_ bicarbonate, PaO_2_ partial pressure of arterial oxygen, PaCO_2_ partial pressure of arterial carbon dioxide, SE standard error, SOFA sequential organ failure assessment, SIPP severity index of paraquat poisoning.

### Predictor of AKI HD

**Figure 1:**
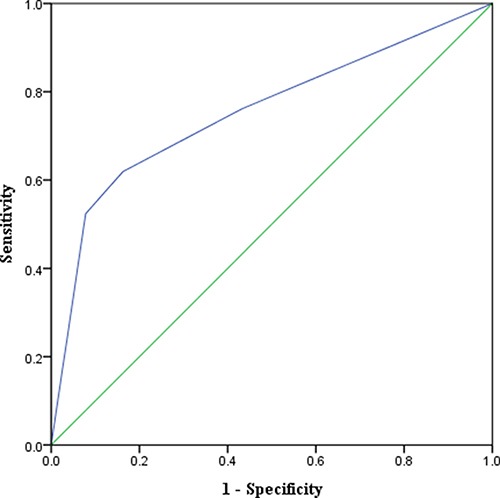
Area under the receiver operating characteristic curve (AUROC) analysis It was revealed that an AKIN_48h_ score ≥ 2 had an AUROC of 0.741±0.068 and predicted AKI HD with a sensitivity of 0.6 and specificity of 0.832.

Multivariate logistic regression analysis indicated that a higher AKIN_48h_ score (*P <* 0.001) was a significant predictor of AKI HD (Table 4). The sensitivity, specificity and cutoff value of this predictor of AKI HD are summarized in Table 5. AUROC analysis revealed that an AKIN_48h_ score = 1.5 was the cutoff point for the prediction of AKI HD. Finally, an AKIN_48h_ score ≥ 2 predicted AKI HD with a sensitivity of 0.6 and specificity of 0.832 (Figure 1).

**Table 4:** Analysis of AKI HD with univariate and multivariate logistic regression models (*n* = 222)

	B	SE	Exp (B)	*P*
Univariate				
Pneumomediastinum	1.744	0.774	5.718 (1.255–26.040)	0.024*
ARDS	1.320	0.495	3.743 (1.418–9.879)	< 0.001***
Creatinine at admission (mg/dL)	0.312	0.105	1.366 (1.113–1.667)	0.003**
AKIN_48h_	0.895	0.213	2.448 (1.613–3.716)	< 0.001***
Nadir AaDO_2_(mmHg)	0.017	0.009	1.017 (1.000–1.035)	0.05
SOFA_48h_	0.241	0.094	1.273 (1.059–1.530)	0.010*
Multivariate				
AKIN_48h_	0.852	0.216	2.344 (1.536–3.577)	< 0.001***

Note: *P < 0.05, **P < 0.01, AKI HD acute kidney injury necessitating hemodialysis, AKIN Acute Kidney Injury Network, ARDS acute respiratory distress syndrome, AaDO_2_ alveolar-arterial oxygen gradient, B beta coefficient or regression coefficient, Exp (B) exponential B or odds ratio, SE standard error, SOFA sequential organ failure assessment

**Table 5:** Sensitivities, specificities and cutoff values of the predictors of AKI and AKI HD (*n* = 222)

	Sensitivity	Specificity	Cutoff value	AUROC ± SE	95% CI	*P*
AKI						
Time to hospital arrival (hours)	0.483	0.740	6.5	0.625 ± 0.041	0.544–0.705	0.003**
SIPP score (hours/mg/L)	0.817	0.432	1.857	0.647 ± 0.043	0.562–0.731	0.001**
PaO_2_ at admission (mmHg)	0.816	0.310	76.7	0.580 ± 0.042	0.498–0.662	0.049*
AKI HD						
AKIN_48h_	0.6	0.832	2	0.741 ± 0.068	0.609–0.874	< 0.001***

Note: *P < 0.05, **P < 0.01, ***P < 0.001, AUROC area under receiver operating characteristic curve, CI confidence interval, SE standard error, AKIN Acute Kidney Injury Network, AKI acute kidney injury, AKI HD AKI necessitating hemodialysis, SIPP severity index of paraquat poisoning.

## DISCUSSION

The present cohort data are important because a large sample was included (*n* = 222), and a standard detoxification protocol was used to treat all paraquat-intoxicated patients: charcoal hemoperfusion, methylprednisolone and cyclophosphamide pulse therapies, and extensive treatment with dexamethasone. As shown in Table 6, the published mortality rates and AKI incidence rates after paraquat poisoning range from 24.0–73.9% and 50.7–100.0%, respectively [2, 3, 10–12, 15–18]. The overall mortality rate in this study was 54.1%, and AKI was common (46.4%) following paraquat ingestion. Some clinically useful variables such as acute hepatitis, the time to hospital arrival, SIPP score and PaO_2_ at admission were powerful predictors of AKI. Finally, an AKIN_48h_ score ≥ 2 predicted AKI HD with a sensitivity of 0.6 and specificity of 0.832.

**Table 6:** Incidence rates of AKI after paraquat intoxication from different hospitals (studies with sample sizes greater than 10)

Study	Year	Area	Sample size	Mortality rate (%)	AKI incidence rate (%)
Current study	2017	Taiwan	222	54.1	46.4
Mohamed et al. [10]	2015	Sri Lanka	50	24.0	76.0
Mohamed et al. [11]	2015	Sri Lanka	66	25.8	56.0
Yang et al. [2]	2012	Taiwan	187	54.5	65.2
Seok et al. [15]	2012	Korea	41	51.2	100.0
Moon et al. [16]	2011	Korea	134	73.9	50.7
Kim et al. [12]	2011	Korea	247	42.9	64.0
Gil et al. [17]	2009	Korea	20	35.0	55.0
Kim et al. [3]	2009	Korea	278	58.8	51.4
Koo et al. [18]	2002	Korea	80	65.0	58.8

Note: AKI acute kidney injury.

Acute hepatitis was a significant predictor of AKI after paraquat intoxication. The mechanisms of paraquat toxicity involve the generation of superoxide anions and the subsequent generation of more harmful reactive oxygen species such as hydrogen peroxide and hydroxyl radical. In addition, nicotinamide adenine dinucleotide phosphate hydrogen (NADPH) is oxidized as it supplies the majority of reducing equivalents to reduce paraquat intracellularly, which thus impairs critical NADPH-requiring biochemical processes [19]. Liver damage has been reported in numerous studies. DeGray et al. [20] proposed that NADPH-cytochrome P-450 reductase reduced paraquat to its radical cations in rat hepatocytes. An additional study [21] evaluated oxidative liver injury 20 hours after oral paraquat intake (a single dose of 150 mg/kg) in rats. Paraquat did not alter the activity of cytosolic superoxide dismutase, but enhanced the activity of mitochondrial superoxide dismutase by 14.0%. Glutathione levels were reduced by 30.0%, and the ratio of glutathione to glutathione disulfide was reduced by 50%. Since mitochondrial superoxide dismutase activity increased while glutathione levels decreased following paraquat intake, the hepatic toxicity of paraquat appeared to be due to the superoxide anion [21].

Since paraquat is eliminated by the kidneys, the ultimate result of paraquat poisoning largely depends on the extent of kidney damage. The nephrotoxicity of paraquat is thought to arise from cycles of reduction and oxidation and the generation of reactive oxygen species [8]. The association between AKI and liver cirrhosis is a well-recognized disease entity [22]. Acute liver failure is frequently accompanied by concurrent AKI as a result of renal hypoperfusion, the nephrotoxicity of the drug itself, or sepsis and systemic inflammation, which causes further morbidity and correlates greatly with a poor prognosis [23]. Nevertheless, our earlier study [2] indicated that while many paraquat patients experienced hepatic complications (46.5%), their toxic hepatitis appeared to be mild and short-lived.

A greater time to hospital arrival and SIPP score were found to predict AKI after paraquat intoxication. This suggested that both the blood paraquat concentration and the time between paraquat ingestion and hospital arrival were significant determinants of AKI. The SIPP score has traditionally been used as a prognostic indicator following paraquat poisoning; a SIP*P* value > 10 is usually associated with mortality [24–26]. We also found that SIPP scores differed significantly between AKI and non-AKI patients (0.7 ± 0.5 vs. 0.5 ± 0.5, *P* = 0.016) and between AKI HD and non-AKI HD patients (0.9 ± 0.4 vs. 0.5 ± 0.5, *P* = 0.001). Additionally, our previous study [5] confirmed that early charcoal hemoperfusion < 4 hours (*P* = 0.020) or charcoal hemoperfusion < 5 hours (*P* = 0.019) significantly reduced the risk of death in severely paraquat-poisoned patients. Similarly, Kim et al. [3] reported that paraquat intoxication was characterized by full-blown AKI after five days, but a return to normal kidney function within three weeks. Furthermore, the risk of mortality in the AKI group was significantly greater than that in the non-AKI group.

A higher PaO_2_ at admission was a significant predictor of AKI after paraquat intoxication. Previously [6], our group found that the respiratory functions and arterial blood gas levels of individuals who had survived paraquat poisoning were slowly restored to more appropriate levels after three months, even when lung fibrosis had occurred. Thus, it appears that severe lung inflammation (rather than pulmonary fibrosis) is the main contributor to lethal hypoxemia when paraquat poisoning is still subacute. Methylprednisolone pulse therapy and dexamethasone are powerful reducers of inflammation. Following dexamethasone therapy, pulse therapy can reduce the profound inflammation resulting from paraquat intoxication; as we previously reported, this enhanced the survival of patients with paraquat intoxication [27–29]. Furthermore, pulse therapy with methylprednisolone can prevent the release of certain factors from lymphocytes which would otherwise stimulate the generation of superoxide anions by macrophages and neutrophils [30]. Steroid treatment can also reduce superoxide production in the arachidonic acid cascade. For these reasons, recurrent pulse therapy with methylprednisolone can stop superoxide from further damaging the lungs and subsequently causing inflammation.

A higher AKIN_48h_ score significantly predicted AKI HD after paraquat intoxication. In our previous study [14], the patient's age, time until hospitalization, circulating level of paraquat, estimated glomerular filtration rate at admission, and SOFA_48h_ score, but not AKIN_48h_ score, significantly predicted death after paraquat intoxication. Nevertheless, Balestracci et al. [31] demonstrated that AKIN stage 3 at admission predicted AKI HD in patients with hemolytic uremic syndrome with a sensitivity of 92% and specificity of 84.2%. Furthermore, Kielstein et al. [32] reported that the majority of patients undergoing interventional lung assist treatment and receiving extended hemodialysis were in AKIN stage 3. Although most studies have reported that an AKIN_48h_ score ≥ 3 predicts mortality [33–35], we found that an AKIN_48h_ score ≥ 2 predicted AKI HD. There was no clear explanation for this, though it may have been due to severe multi-organ damage following large-dose exposure to paraquat.

ARDS was a significant predictor of AKI HD in univariate analysis, but not after multivariate analysis. ARDS was independently associated with AKI in critically ill patients [36]. A high percentage (34.0%) of patients undergoing interventional lung assist treatment received extended hemodialysis for AKI [32]. When ARDS concurs with AKI, the duration of stay in the intensive care unit and use of resources both increase, and the mortality rate markedly increases to nearly 60% [37]. Recent studies have demonstrated that ARDS can cause AKI, and AKI can alter leukocyte trafficking and pulmonary vascular permeability [37]. The lungs and kidneys receive a high circulating volume of blood every minute. The lungs are exposed to the entire cardiac output, and the kidneys are exposed to 22.0% of the cardiac output. Therefore, during paraquat intoxication, both organs are exposed to large amounts of paraquat and the accompanying circulating inflammatory mediators, neutrophils and monocytes, and are thus at risk for injury [38].

Similarly, pneumomediastinum was a significant predictor of AKI HD in univariate analysis, but not after multivariate analysis. This may be because too few paraquat patients (only 8/222) suffered from pneumomediastinum to reveal a significant impact on AKI HD. The Macklin effect may explain why paraquat-intoxicated patients develop pneumomediastinum [39]. Paraquat poisoning results in stiff lung and a predisposition to barotrauma. After the alveoli have ruptured, free air may travel toward the hilum of the lung via peribronchial vascular sheaths, and then spread proximally to the mediastinum. Zhou et al. [40] reported that pneumomediastinum occurred in 21.3% of their paraquat-intoxicated patients (16/75). Thirteen of these patients experienced pneumomediastinum within three days of consuming paraquat, and 15 died within three days of the onset of pneumomediastinum. Toxic hepatitis (*P* = 0.008), respiratory insufficiency (*P* = 0.003) and acute renal failure (*P* = 0.001) occurred more frequently in patients with pneumomediastinum than in those without. Finally, pneumomediastinum was found to predict a greater risk of 90-day (*P* = 0.045) and 5-day mortality (*P* = 0.017) [40].

In summary, AKI was common (46.4%) following paraquat ingestion, and certain clinically useful variables such as acute hepatitis, the time to hospital arrival, SIPP score and PaO_2_ at admission were powerful predictors of AKI. A higher AKIN_48h_ score significantly predicted AKI HD after paraquat intoxication. Nevertheless, as this study was retrospective, included a small population of patients and involved a short period of follow-up, further studies are warranted to confirm our results.

## MATERIALS AND METHODS

### Ethics statement

This retrospective observational study complied with the guidelines of the Declaration of Helsinki and was approved by the Medical Ethics Committee of Chang Gung Memorial Hospital. Since this study involved the retrospective review of existing data, approval from the Institutional Review Board was obtained, but without specific informed consent from patients. Furthermore, not only were all data securely protected (by the delinking of identifying information from the main data sets) and made available only to the investigators, but they were also analyzed anonymously. Finally, all primary data were collected according to procedures outlined in epidemiology guidelines to strengthen the reporting of observational studies.

### Patients

In total, 222 patients were referred for the management of intentional paraquat ingestion between 2000 and 2012.

### Diagnosis of paraquat poisoning

A presumptive diagnosis of paraquat poisoning was based on exposure history, clinical effects, and physical and laboratory examinations, especially the urine sodium dithionite screening test. The urine sodium dithionite reaction depends on the reduction of paraquat by sodium dithionite under alkaline conditions to form stable, blue-colored radical ions [41]. The generation of a strong navy or dark blue color generally indicates significant paraquat ingestion and often forebodes a poor prognosis. The urine test was used as a paraquat screen, while a confirmatory diagnosis of paraquat poisoning was only possible through the analysis of the blood paraquat concentration (spectrophotometry, Hitachi, Tokyo, Japan). At our hospital, the result of a urine paraquat test is available in 30 minutes, but the result of a blood paraquat test is not available for at least 4 hours.

### Definition of AKI

The definition of AKI was based on the Acute Kidney Injury Network (AKIN) score [42].

### Definition of acute lung injury and acute respiratory distress syndrome (ARDS)

Acute lung injury is a continuum of clinical and radiographic changes affecting the lungs, characterized by acute-onset severe hypoxemia, unrelated to left atrial hypertension, occurring at any age. At the severe end of this spectrum lies ARDS. ARDS was defined according to the American-European consensus conference as an acute onset of bilateral pulmonary infiltrates, a ratio of partial pressure of arterial oxygen (PaO_2_) to fraction of inspired oxygen ≤ 200 mmHg, and a pulmonary artery occlusion pressure ≤ 18 mmHg or absence of left atrial hypertension [43].

### Definition of acute hepatitis

Acute hepatitis was defined if the serum level of alanine aminotransferase (ALT) was > 72 U/L (normal 0–36 U/L), or total bilirubin was > 1.5 mg/dL (normal 0–1.3 mg/dL) [2].

### Inclusion and exclusion criteria

Patients were included in this study if they were > 18 years of age and had urine paraquat tests that displayed dark or navy blue coloring (> 5 ppm). Patients were excluded from the study if the paraquat exposure was dermal or intravascular [44, 45]. Patients were also excluded if paraquat was not detectable in their urine and blood or if they had major comorbidities, such as cancer or heart, lung, renal, or liver diseases. Diagnoses of major comorbidities were based on detailed clinical, physical, and laboratory examinations. Patients with pre-existing serum creatinine concentrations > 1.2 mg/dL, ALT concentrations > 36 mg/dL, or total bilirubin concentrations > 3 mg/dL were also excluded.

### Sequential organ failure assessment (SOFA) and AKIN scores

The following data were collected: baseline demographics, SOFA and AKIN scores 48 hours after admission (SOFA_48h_ and AKIN_48h_), and the time to ARDS (ARDS patients) or nadir PaO_2_ (non-ARDS patients). The SOFA score consists of six variables, each representing an organ system. Each organ system is assigned a point value from 0 (normal) to 4 (high degree of dysfunction/failure) [14]. The AKIN criteria classify AKI into three stages of severity (stages 1, 2, and 3) [14].

### Severity index of paraquat poisoning (SIPP) score

The SIPP score is calculated as the time to treatment (hours) x serum paraquat concentration (ppm) [24].

### Protocol for paraquat detoxification

The paraquat detoxification protocol [4, 5] included gastric lavage with a large amount of 0.9% saline, followed by 1 g/kg activated charcoal and 250 mL magnesium citrate through a nasogastric tube. Charcoal hemoperfusion with a charcoal-containing (Adsorba, Gambro, Germany) dialysis machine (Surdial, Nipro, Japan) was initiated if the urine paraquat concentration was > 5 ppm [5]. A second session of hemoperfusion was arranged if the urine paraquat concentration was > 5 ppm 4 hours after the first hemoperfusion. The protocol also included pulse therapies of cyclophosphamide (15 mg/kg/day) for two days and methylprednisolone (1 g/day) for three days [4]. Intravenous dexamethasone (20 mg/day) was administered for another 11 days after methylprednisolone pulse therapy. Since cyclophosphamide and methylprednisolone were potentially removable by charcoal hemoperfusion, both agents were administered after the extracorporeal treatment. Pulse therapies with cyclophosphamide and methylprednisolone were repeated if the PaO_2_ was < 60 mmHg and the duration was more than two weeks after the initial treatment, unless the patient had leucopenia (white cell count < 3000/m^3^). Finally, all patients received normal inspired oxygen therapy (FiO_2_ 21%) throughout their hospitalization. The rationale for normal inspired oxygen therapy was that an increased FiO_2_ could increase oxidative stress, and the production of free radicals and superoxide might worsen the acute lung injury and systemic toxicity [46].

### Statistical analysis

Data are expressed as the mean ± standard deviation or number (percentage), unless otherwise stated. All variables were tested for normal distribution by the Kolmogorov-Smirnov test. Student's *t* test was used to compare the means of continuous variables and normally distributed data. The Mann-Whitney *U* test was used for non-normally distributed data. Categorical data were analyzed with the chi-square test. Finally, risk factors were assessed by univariate logistic regression analysis, and variables that were statistically significant (P<0.05) were included in a multivariate analysis; multiple logistic regression was applied based on forward elimination of data. Calibration was assessed with the Hosmer-Lemeshow goodness-of-fit test to compare the number of observed and predicted AKI and HD cases in risk groups for the entire range of AKI and HD probabilities. Discrimination was assessed by analysis of the area under the receiver operating characteristic curve (AUROC). The AUROCs were compared by a non-parametric approach. AUROC analyses were also used to calculate cutoff values, sensitivities, specificities, and overall correctness. Finally, the cutoff points were calculated in accordance with the best Youden index (sensitivity + specificity - 1). All statistical tests were two-tailed, with *P* values < 0.05 being considered statistically significant. Data were analyzed with SPSS 12.0 software for Windows (SPSS, Inc., Chicago, IL, USA).
